# Editorial: Polycomb group (PcG) proteins in development and disease

**DOI:** 10.3389/fcell.2024.1361468

**Published:** 2024-02-27

**Authors:** Prasad Pethe, Satyanarayana M. R. Rao, Simone Tamburri, Raghuvir Singh Tomar

**Affiliations:** ^1^ Symbiosis Centre for Stem Cell Research (SCSCR), Symbiosis International (Deemed University), Pune, India; ^2^ Jawaharlal Nehru Centre for Advanced Scientific Research, Bengaluru, India; ^3^ European Institute of Oncology (IEO), Milan, Italy; ^4^ Indian Institute of Science Education and Research, Bhopal, India

**Keywords:** polycomb group proteins, epigenetics (chromatin remodeling), lineage specifications, gene expression, diseases

## Introduction

The Polycomb group (PcG) proteins are epigenetic modulators that add histone modifications at specific locations to bring about a change in gene expression and have been shown to be required for lineage specification and organ homeostasis. PcG proteins such as RING1B and MEL18 help in X chromosome inactivation in females during early development; while EZH2 regulates the proliferation of epidermal and intestinal stem cells, thereby maintaining tissue homeostasis. In cancer pathobiology PcG proteins such as EED, BMI1 and EZH2 have been implicated in breast, prostate, colon, pancreatic and other cell type cancer etc. Thus, Polycomb group (PcG) proteins play a major role in both development and disease. However, there are several aspects of Polycomb group (PcG) mediated gene regulation that remain to be uncovered. In this Research Topic, we have brought together the recent advances in terms of the role of PcG proteins in development and disease.

## PcG proteins in development and disease

Brain tissue is one of the most complex tissue, composed of different kinds of neuronal cells with each cell exhibiting unique gene expression profile. Epigenetic mechanisms allow for this differential gene expression for normal brain functioning, but in some cases when the epigenetic mechanisms including PcG regulation fail it may lead to disrupted functions. Peedicayil through an opinion article, presented the available literature on complex psychological disorders like schizophrenia and anxiety. The opinion enumerates several examples of PcG protein misregulation that affect neuronal functions in various *in vitro* and *in vivo* studies. Further, it proposes that PcG proteins could be misregulated in several other neurological disorders, emphasizing that more work is needed in this area.

Diabetes is another complex disease characterized by eventual loss of the pancreatic β-cells and understanding the development of pancreatic β cell at the molecular level will give us leads which could be used to treat diabetes. EZH2 catalyzed H3K27me3 histone modification at specific gene promoters has been shown to play a critical role in differentiation of progenitor cells into pancreatic or hepatic cells during murine development (Xu et al., 2011). Later, d using inducible knockout system for Ring1b, it was demonstrated that Ring1B pioneers repression of genes in pancreatic cells, however in adult β cells the repression mechanisms shifts to Ring1b independent mechanism van Arensbergen et al., 2013; Varghese and Dhawan, build on the role of PcG proteins defined in murine pancreatic β cell development to bring forth some important questions about their role in human pancreatic β cell development, maturation and senescence, in normal and diabetic condition.

It has become increasingly clear that 3D genome organization plays a critical role gene expression. Techniques such as 3C, Hi-C and 4C have demonstrated that the numerous protein complexes assist in DNA looping and this allows transcriptional machinery to access regions of DNA that are far apart; thus these newer techniques have helped us to better understand the importance of protein complexes such as PcGs in 3D DNA arrangement. Guo and Wang, bring together evidence from reports from embryonic stem cells and early embryo development, which show that the Polycomb proteins form PRC condensates via the liquid-liquid phase separation (LLPS), this aids in chromatin silencing. The review a poses a paradox: PcG proteins also perform gene activation role besides their repressive role and this merits further investigation.

Mutations in some of the PcG proteins such as BMI1, EZH2, SUZ12, and EED are known to result in cancers of various tissues (Chan and Morey, 2019; Doyle et al., in their review highlight that 3D genome organization might be controlled by variant PcG proteins within the large multimeric PcG protein complexes. Authors discussed various PcG interacting epigenetic modifiers such as BAP1, ASXL, NSD1 and DNMTs which may alter the 3D genome organization and this merits further research. PcG proteins and associated proteins may get disrupted and this could further lead to cancer formation. The review discusses that, despite a number of studies showing that several cancers can arise due to mutations in PcG proteins, and availability of drugs that can regulate the PcG protein activity, it is surprising that there are very few drugs that are approved for use for cancer treatment. Hence there is an urgent need to develop novel small molecules that can target PcG proteins for cancer treatment.

## Conclusion

The articles published in this Research Topic showcase the role of Polycomb group (PcG) protein in regulating several developmental genes and impact on the developmental trajectory and later functioning. The PcG proteins assemble into large protein subunits that regulate the higher 3 dimensional structure of the DNA and thereby execute transcription.
